# Recombination plasticity in response to temperature variation in reptiles

**DOI:** 10.1371/journal.pgen.1011772

**Published:** 2025-08-04

**Authors:** Laura González-Rodelas, Cristina Marín-García, Clara Romero, Gala Pujol, Laia Marín-Gual, Lukáš Kratochvíl, Aurora Ruiz-Herrera

**Affiliations:** 1 Departament de Biologia Cel·lular, Fisiologia i Immunologia, Universitat Autònoma de Barcelona, Cerdanyola del Vallès, Spain; 2 Genome Integrity and Instability Group, Institut de Biotecnologia i Biomedicina, Universitat Autònoma de Barcelona, Cerdanyola del Vallès, Spain; 3 Department of Ecology, Faculty of Science, Charles University, Prague, Czech Republic; University of Wisconsin–Madison, UNITED STATES OF AMERICA

## Abstract

The survival of species depends on their ability to adapt to environmental changes. While organisms are known to activate common transcriptional pathways in response to temperature variations, the impact of temperature on recombination, a key source of genetic variability, remains largely unexplored. Previous studies in model species have shown that the frequency of recombination during meiotic prophase I can be influenced by extreme temperatures. Yet, it remains unclear whether this effect is also conserved in non-model vertebrates. In this study, we investigated the effect of temperature on recombination in the Guibé’s ground gecko (*Paroedura guibeae)*, an ectotherm species. We analyzed the formation of double-strand breaks (DSBs) and crossovers (COs) by immunolocalizing the meiotic proteins involved in these processes. Furthermore, we determined the frequency and chromosomal location of COs and the levels of CO interference (COI). Our findings show the presence of hyper-COs spermatocytes in individuals exposed to both high and low temperatures. Notably, this significant increase in COs was associated with a decrease in chromosome axis lengths and elevated levels of meiotic DSBs in later stages of prophase I. In conclusion, our results provide new insights into the effects of environmental temperatures on meiotic recombination in ectothermic species, underscoring the intricate interplay between environmental factors and genetic processes.

## Author summary

Species survival hinges on adapting to environmental changes. While organisms activate common genetic pathways in response to temperature shifts, the impact on recombination—a key source of genetic diversity—remains largely unexplored, especially in non-model vertebrates. Here we investigated how temperature affects recombination in the Guibé’s ground gecko, an ectothermic species. By analyzing proteins involved in double-strand breaks (DSBs) and crossovers (COs), we uncovered a significant increase in COs upon exposed to high and low temperatures, linked to shorter chromosome axis lengths and higher levels of meiotic DSBs. Our findings reveal the complex interplay between temperature and genetic processes in ectothermic species, offering new perspectives on how environmental factors shape genetic diversity.

## Introduction

As biodiversity is experiencing a rapid decline, dramatically enhanced by climate-related environmental shifts, understanding the mechanisms of species adaptation to external stimuli and their effects on individual reproduction and survival is key. This is especially true for ectothermic vertebrates, including fishes, amphibians and reptiles, which are particularly vulnerable to climate change and pollution. Variations in air, water and soil temperatures can significantly impact their basking behavior and embryonic development [1,2].

In this context, deciphering the role of genome regulation in species adaptation to changing environments is fundamental. Alterations in genome architecture in response to external stimuli have been suggested to trigger swift changes in gene expression [3]. Among the environmental stimuli that can influence genome regulation and function, temperature is the most pervasive. It affects a myriad of processes, including sex determination [4], meiosis and recombination [5–7]. As such, recent studies have suggested that changes in recombination rates can facilitate adaptation in an intermittently fluctuating environment [8]. This underscores the importance of understanding the genomic plasticity of recombination in the context of climate change.

Recombination, a pivotal process generating genetic variability in gametes, is a cornerstone for evolutionary adaptation. It begins in early stages of meiosis, with the controlled generation of hundreds of double-strand breaks (DSBs) across the genome by a highly conserved endonuclease, SPO11 [9,10]. These DSBs facilitate homology search and synapsis of homologous chromosomes through homologous recombination, involving proteins like RPA, RAD51 and DMC1, among others (reviewed in [11]). DSBs destined to become crossovers (COs) are resolved by the repair pathway directed by MSH4/MSH5 and MLH1/MLH3 [12–16]. In most organisms, the initial number of DSBs greatly exceeds the number of COs indicating that the majority of DSBs are resolved as non-COs (NCOs) [17–19]. In the case of human and mice, for example, 10% of DSBs are repaired as COs [19–21]. The assurance of COs events is tightly regulated by homeostatic mechanisms that modulate the ratio between COs and NCOs [18].

In most species, COs are not randomly distributed throughout the genome but concentrated in recombination hotspots [22–26]. The distribution of these hotspots is influenced by factors at both the chromosomal and individual levels, including chromosome size and reorganizations, as well as sex-specific patterns [22,24,27–34]. COs are characterized by three primary attributes: (i) they occur within distinct genomic regions (hotspots), (ii) a minimum of one CO event is guaranteed for each chromosome pair, facilitating the correct disjunction of homologous chromosomes, and (iii) they exhibit a phenomenon known as ‘interference’, where COs tend to be distributed in a uniformly spaced manner across the genome. However, not all COs are subject to interference. This distinction has led to the classification of COs into two types: class I (interfering), which are Msh4–Msh5-dependent and follow a regulated distribution, and class II (non-interfering), which are Mus81–Mms4-dependent and occur independently of one another [35–38]. Additionally, centromeric regions often reflect CO inhibition (the so-called centromere effect [39–41]). Evidence also suggests a complex interplay between the length of meiotic chromosome axes and the extent of CO interference (COI), though the nature of this phenomenon is still under discussion [11,42].

The effect of temperature on meiotic recombination has been known since early experiments in *Drosophila* and plants [43–45]. These studies identified three major temperature-induced changes in: (i) chiasmata frequency, (ii) distribution of chiasmata along chromosome and (iii) internal coiling (how the DNA coils around itself) [45]. Different species exhibit distinct meiotic temperature tolerances, with plants generally showing higher thermal tolerance ranges than animals (reviewed in [5]). Experimental evidence from genetic maps or cytological description of COs in animals (flies and worms) and plants (crops and wild species) has revealed different trends in temperature-induced changes in recombination: (i) steady decreases, where recombination rate decreases continuously with increasing temperature; (ii) steady increases, where recombination rate increases steadily with increasing temperature; (iii) U-shaped curves, where recombination is highest at low and high temperatures and lower at moderate temperatures; (iv) inverted U-shaped curves, where recombination decreases at low and high temperatures; and (v) no effect, where temperature has no significant effect on the recombination rate [5,46,47]. Among these patterns, many species studied to present U-shaped curves, meaning that midrange temperatures correspond to the lowest recombination rate, and both increases and decreases in rearing temperature are associated with elevated recombination [5,46,47]. However, it remains unclear whether the effects of low and high temperatures are mechanistically distinct.

Within this framework, geckos (Gekkota — Reptilia) emerge as an interesting model for studying recombination and sex determination and responses to environmental stimuli, as this group exhibits both Temperature Sex Determination (TSD) as well as Genotypic Sex Determination (GSD) with XX/XY and ZZ/ZW sex chromosomes [4,48,49]. Research on temperature-induced recombination changes in reptiles is scarce, with studies confined to the Australian gecko *Phyllodactylus marmoratus*, which show annual cycles in CO number in nature, with the lowest rates observed when temperatures are the highest [50]. Here we examine the effect of temperature on meiotic recombination in the Guibé’s ground gecko (*Paroedura guibeae*), a saxicolous (living among rocks) species from Madagascar, with an estimated optimal physiological temperature of 27 °C [51]. This species is typified by a karyotype of 2n = 34 chromosomes [52], with all acrocentric chromosomes except for chromosomes 1 and 4, which are metacentric [49]. Our analysis of DSBs and COs dynamics through the immunolocalization of meiotic proteins reveals a U-shaped recombination frequency curve, with minimum CO levels at 24–28 °C. We also observed hyper-CO spermatocytes at the highest (30 °C) and lowest (20 °C) temperatures tested, linked to increased meiotic DSBs in later stages of prophase I. Moreover, we detected a decrease in CO interference, resulting in a chromosomal redistribution of COs. All in all, our analysis underscores the importance of studying recombination in the context of temperature variation, which is essential for understanding the mechanisms that drive species adaptation and evolutionary processes.

## Results

### CO frequencies follow a U-shaped curve

**Fig 1:**
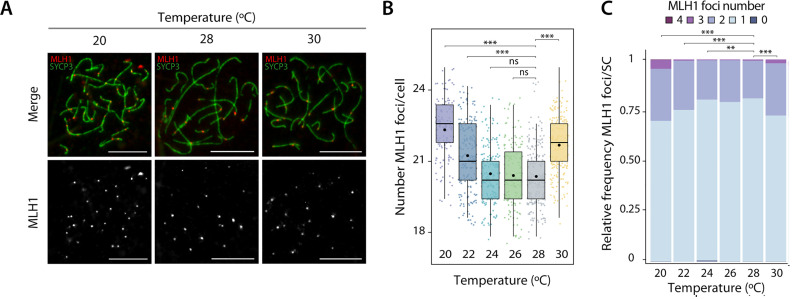
Crossover formation at pachytene stage of prophase I. **(A)** Representative immunofluorescence images of spermatocyte spreads labeled with antibodies against SYCP3 (green) and MLH1 (red) for individuals treated at 20 °C, 28 °C and 30 °C. Scale bar: 10 μm. **(B)** Plot representing the number of MLH1 foci per cell for each temperature, with median values (center line), mean values (dot), and standard deviation (± SD). A total of 1,169 cells were analyzed across six temperatures: n = 175 cells for 20 °C, n = 207 cells for 22 °C, n = 186 cells for 24 °C, n = 147 cells for 26 °C, n = 230 cells for 28 °C and n = 224 cells for 30 °C. Asterisks represent statistically significant values between temperatures (T-test **p-value < 0.01 and ***p-value < 0.001; ns: non-significant). **(C)** Relative frequency plot showing the number of MLH1 foci (0,1, 2, 3 or 4) per synaptonemal complex (SC) for each temperature. Asterisks represent statistically significant values when compared to the physiological condition (28 °C) (Chi-squared test, *p-value < 0.05, **p-value < 0.01 and ***p-value < 0.001).

We first examined whether meiotic recombination was affected by temperature variation in the gecko *P. guibeae*. To this end, we scored the number of MLH1 (a proxy of interfering COs) foci per cell at the pachytene stage as an indicator of recombination frequency (Fig 1A). A total of 1,169 spermatocytes from 12 animals exposed to six different temperatures (20 °C, 22 °C, 24 °C, 26 °C, 28 °C and 30 °C) were analyzed. To capture inter-individual variability, two animals were analyzed for each temperature. Since no differences were found between the number of MLH1 per cell and individual (T-test, p-value > 0.05, S1 Fig), data from individuals subjected to the same temperature were pooled for subsequent analyses.

Considering number of MLH1 foci per cell observed at 28 °C (20.18 ± 1.52) a condition close to the species’ physiological optimum [51], we observed a significant increase in the number of MLH1 foci per cell at both high (30 °C) and low temperatures (20 °C and 22 °C) (Fig 1B). Values ranged from 22.93 ± 1.61 at 20 °C to 21.30 ± 1.74 at 22 °C, and 21.86 ± 1.47 at 30 °C (T-test, p-value < 0.001; Fig 1B). Conversely, no significant differences in CO frequency were observed between 24 °C and 26 °C (20.34 ± 1.44 and 20.24 ± 1.71, respectively) (T-test, p-value > 0.05) compared to 28 °C. These results indicate the presence of hyper-CO spermatocytes at both high and low temperatures, indicating that COs frequencies follow a U-shaped curve in *P. guibeae*.

Since each pair of homologous chromosomes requires at least one CO to ensure proper chromosome segregation (and hence fertility) during the first meiotic division (the so-called obligatory CO [53,54]), we quantitatively assessed whether the number of MLH1 foci (0, 1, 2, 3 or 4) per synaptonemal complex (SC) was altered at different temperatures. We found that the frequency of SCs with zero MLH1 foci was consistently low across all temperatures tested (from 0.08% at 22 °C to 0.6% at 24 °C), with no statistical differences observed (Chi-squared test, p-value > 0.05) (Fig 1C). However, when considering one or more MLH1 foci per chromosome, differences were detected at both high (30 °C) and low temperatures (20 °C and 22 °C) (Chi-squared test, p-value < 0.001) (Fig 1C). The proportion of SCs with one CO (average frequency value of 72.3%) was significantly lower at the lowest (20 °C and 22 °C) and highest (30 °C) temperatures tested, when compared to temperatures close to the physiological optimum (24 °C, 26 °C and 28 °C) (average frequency value of 79.9%) (Chi-squared test, p-value < 0.05). This resulted in a higher proportion of SCs with two or more COs at extreme temperatures tested (average frequency of 27.4%) compared to temperatures near the physiological optimum (average frequency of 19.7%) (Chi-squared test, p-value < 0.001) (Fig 1C).

### Long chromosome axes experience a larger shift in CO number

**Fig 2:**
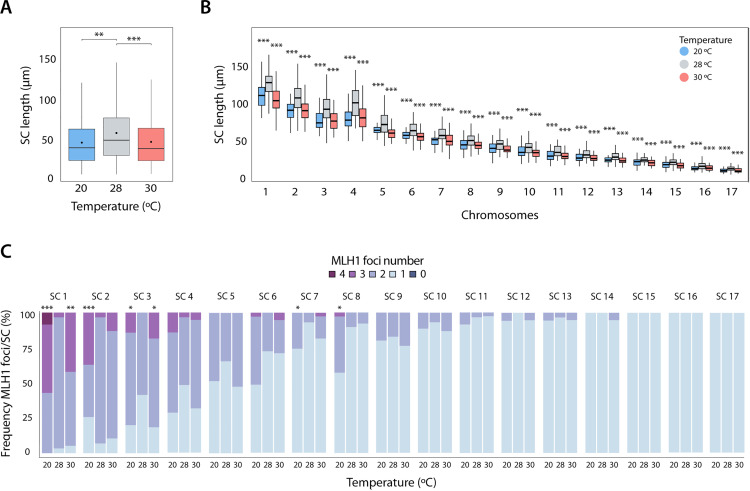
SC axis length and chromosomal distribution of crossovers. **(A)** Representation of the synaptonemal complex (SC) size. Boxplots display the SC size with median values (center line), mean values (dot), and standard deviation (± SD). A total of 145 cells were analyzed across three temperatures: 20 °C (n = 50 cells), 28 °C (n = 45 cells), and 30 °C (n = 50 cells). Asterisks represent statistically significant values between temperatures (T-test, **p-value < 0.01, ***p-value < 0.001). **(B)** Boxplots represent the SC size for each chromosome (Tukey HSD test, ***p-value < 0.001). *P. guibeae* has predominantly acrocentric chromosomes, except for chromosomes 1 and 4, which are metacentric. **(C)** Frequency plots showing the number of MLH1 foci (0,1, 2, 3 or 4) per SC according to size for three temperatures: 20 °C, 28 °C and 30 °C. A total of 145 spermatocytes were analyzed: n = 50 cells for 20 °C, n = 45 cells for 28 °C and n = 50 cells for 30 °C. Statistical analyses were performed using the Chi-squared test, comparing each temperature with the control (28 °C) (*p-value < 0.05, **p-value < 0.01, ***p-value < 0.001)*. P. guibeae* has predominantly acrocentric chromosomes, except for chromosomes 1 and 4, which are metacentric.

We next examined whether temperature influenced chromosome CO distribution and SC axis length (Fig 2). To this end, we measured the length of autosomal SCs and analyzed the chromosomal distribution of MLH1 foci at three temperatures, the one closer to optimal physiological temperature (28 °C), and the lowest (20 °C) and the highest (30 °C) temperatures tested where significant effects were detected.

Chromosome axis length of meiotic chromosomes at pachytene can be accurately measured by immunostaining SC components (i.e., SYCP3) in spread nuclei (e.g., [29,31]). Only spermatocytes at the pachytene stage with well-defined SCs were scored. Then, SCs were ranked according to their relative lengths and centromere positions (SCs 1–17), established by immunofluorescence using an antibody to detect centromeric proteins (see Methods). The lengths of SCs scored at 28 °C were used as a reference to categorize them into large (SCs 1–4 ranging from 130.48 to 111.86 µm in length) and short (SCs 5–17 ranging from 80.60 to 17.33 µm in length) chromosomes (S1 Table).

Our results revealed that exposure to both low (20 °C) and high (30 °C) temperatures resulted in shorter chromosome axes when compared to 28 °C (T-test, p-value < 0.01) (Fig 2A). Average values of SCs lengths per cell ranged from 48.49 µm at 30 °C to 48.31 µm at 20 °C when compared to 60.31 µm at 28 °C (Fig 2A and 2B and S1–S3 Tables). These differences in SC lengths were accompanied by a higher number of MLH1 foci per axis in long SCs (Fig 2C). Specifically, when analyzing the relative frequency of MLH1 foci (0, 1, 2, 3 or 4) per SC, the excess of COs at 20 °C and 30 °C was concentrated in long chromosomes (SCs 1–4) (Chi-squared test, p-value < 0.05) (Fig 2C). For instance, in SC 1, significant differences were detected at both 20 °C and 30 °C temperatures when considering more than two MLH1 foci per chromosome (average frequency value of 57.14% at 20 °C and 42.1% at 30 °C) when compared to 28 °C (average value of 3.45%) (Chi-square test, p-value < 0.001). Fewer than two COs tended to accumulate in short SCs, so changes occur preferentially in longer SCs. Moreover, we observed that CO density along SCs increased as axis length decreased, a pattern previously reported for mammalian species [29]. This trend was observed across all three temperatures analyzed (S1–S3 Tables).

**Fig 3:**
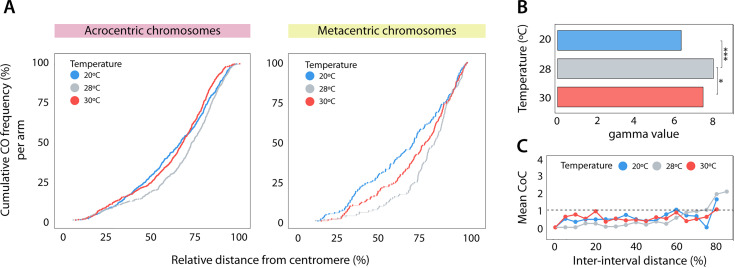
Crossover interference analysis. **(A)** Cumulative frequency plots of COs for acrocentric bivalents and metacentric chromosomes considering all MLH1 foci per chromosomal arm analyzed (Kolmogorov-Smirnov test, p-value < 0.01, p-value < 0.001). **(B)** Shape parameter of gamma distribution. Data represents gamma values between the first and second MLH1 foci in chromosomes with two MLH1 foci for all temperatures. Asterisks represent statistically significant values between temperatures (Tukey HSD test, *p-value < 0.05, ***p-value < 0.001). **(C)** Coefficient of coincidence (CoC) curve indicates the quantification of crossover interference (COI). The horizontal line represents the expected level without interference (mean CoC = 1). For all plots, a total of 140 spermatocytes were analyzed: n = 50 cells for 20 °C, n = 40 cells for 28 °C and n = 50 cells for 30 °C.

We next calculated the position of individual MLH1 foci for each SC using the centromere as a reference. For comparison among chromosomes and temperatures, the MLH1 position was scored as the relative position of each CO to the length of the chromosome, with each SC divided into 10% intervals (see Methods). We detected that the position of MLH1 foci along the SC length differed among temperatures when considering both acrocentric (Kolmogorov-Smirnov test, p-value < 0.001) and metacentric chromosomes (Kolmogorov-Smirnov test, p-value < 0.01) (Fig 3A). When plotting all MLH1 foci in acrocentric chromosomes, COs tend to localize further away from the centromere, between the 75% and 100% of SC length at 28 °C. This differs from the observed at both low (20 °C) and high (30 °C) temperatures, where COs localized preferentially towards more internal regions (between 25% and 75% of SC length). A similar trend was noted in metacentric chromosomes, with COs shifting towards the internal SC regions at both low and high temperatures (Fig 3A). In all cases, we detected suppression of recombination at the vicinities of the centromere (the so-called centromere effect [55]), although this suppression was less pronounced at both low (20 °C) and high (30 °C) temperatures (Fig 3A).

Given that longer chromosome axes exhibited a more significant shift in CO numbers per cell (Fig 2C), we explored whether these differences affected CO chromosomal distribution. Our analysis revealed variations in CO distribution across chromosomes at both low and high temperatures, particularly in larger chromosomes (T-test, p-value < 0.01) (S4 Table). Specifically, chromosomes SCs 1–4 showed differences in CO distribution at 20 °C and 30 °C, with average values of 58.6% and 61.1% of chromosomal axis length, respectively, compared to 28 °C, which had an average value of 69.2% (T-test, p-value < 0.01). In contrast, shorter chromosomes (SCs 5–17) consistently exhibited more distal CO positions at low and high temperatures, localizing at 65.65% of SC length (T-test, p-value > 0.05) (S4 Table). Therefore, we confirm that longer chromosomes tend to accumulate the excess temperature-induced COs, leading to a redistribution of COs along the SC axes.

### CO interference decreases at both high and low temperatures

Given the observed changes in CO distribution, we further explored whether crossover interference (COI) could explain our findings. COI refers to the phenomenon where adjacent COs on the same chromosome occur farther apart than would be expected by random distribution [42]. In this scenario, the presence and intensity of COI, represented by the interference parameter gamma (ɣ), can be effectively modelled by the gamma distribution, which describes how recombination sites are distributed along chromosomes. A higher value of ɣ corresponds to a more widely spaced distribution of COs and a stronger presence of COI. Specifically, ɣ = 1 indicates the absence of COI [56]. We observed that gamma values differed significantly among temperatures (Tukey HSD test, p-value < 0.05) (Fig 3B). Samples subjected to 28 °C exhibited a higher level of COI (ɣ = 8.35) compared to those at low (20 °C, ɣ = 6.51) and high temperatures (30 °C, ɣ = 7.66) (Fig 3B and S5 Table). This indicates shorter distances between COs and weaker COI at the extreme temperatures tested.

COI was further quantified by analyzing the coefficient of coincidence (CoC) [57], which measures the frequency of double CO events relative to what would be expected if they were independent. Values close to 0 indicate high COI, while values close to 1 suggest reduced COI [51,55]. The CoC curve confirmed a decrease in COI at both 20 °C and 30 °C (Fig 3C). Our analysis showed that individuals exposed to both high and low temperatures, COI disappeared (CoC = 1) when the distance between two MLH1 foci was at least 60% of the chromosome length. In contrast, at 28 °C, interference vanished when the distance was more than 70% of the SC, suggesting that interference operated over a longer distance.

### High and low temperatures drive an accumulation of DSB at late stages of prophase I

**Fig 4:**
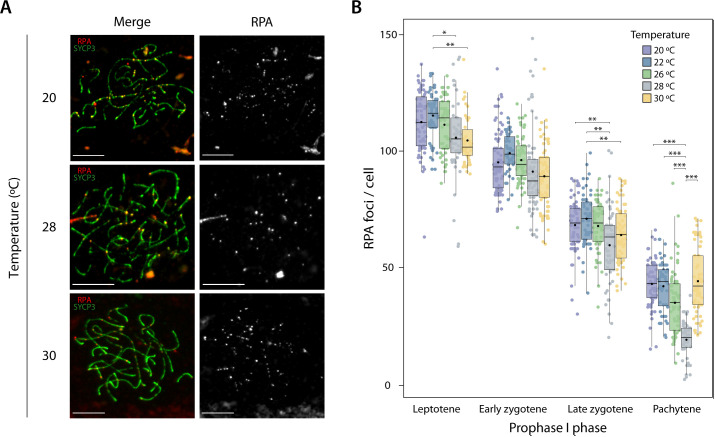
Double-strand break formation dynamics during prophase I. **(A)** Representative immunofluorescence images of pachytene spermatocyte spreads labeled with antibodies against SYCP3 (green) and RPA (red) for individuals treated at 20 °C, 28 °C and 30 °C. Scale bar: 10 μm. **(B)** Plot representing the number of RPA foci per cell detected at leptotene, early zygotene, late zygotene and pachytene for each condition. Boxplots are presented as median values (center line); mean values (dot) ± SD. A total of 1,195 cells were analyzed, distributed across five temperatures: n = 266 cells for 20 °C, n = 208 cells for 22 °C, n = 233 cells for 26 °C, n = 218 cells for 28 °C, and n = 270 cells for 30 °C. Asterisks represent statistically significant values between temperatures (Tukey HSD test, *p-value < 0.05, **p-value < 0.01 and ***p-value < 0.001).

Given the variability in COs numbers and distribution detected between temperatures, we investigated whether a similar pattern was observed for proteins involved in the formation of DSBs in early stages of prophase I. Replication protein A (RPA), along with other proteins, associates with single-stranded DNA (ssDNA) following DSB formation and accumulates at these sites [58]. Furthermore, RPA is thought to associate with later recombination intermediates, such as the ssDNA freed during D-loop formation [59]. By analyzing the number of RPA sites in early prophase I, the number of DSBs formed during the early stages of meiosis can be estimated. To that aim, the dynamics of DSBs formation during meiotic prophase I was investigated through the immunodetection of the RPA repair protein together with the lateral element of the SC (SYCP3 protein) (Fig 4A). A total of 1,195 spermatocytes were analyzed for the calculation of the number of RPA foci per each cellular stage and temperature (Fig 4B).

Consistent with known meiosis dynamics [60], a decrease in the number of RPA foci per cell was observed as meiotic prophase I progressed, peaking in leptotene (112.21 ± 26.75 at 20 °C, 105.55 ± 32.57 at 28 °C, and 104.35 ± 27.5 at 30 °C) and reaching a minimum in pachytene (42.92 ± 31.04 at 20 °C, 19 ± 30.35 at 28 °C, and 44.10 ± 27.39 at 30 °C) (Fig 4B). The mean number of RPA foci at leptotene at 28 °C was used as a proxy for the overall number of DSBs produced in the early prophase I in conditions circa to the physiological optimum. The same trend of progressively decreasing RPA foci as prophase I progresses was also observed in the different temperatures tested (Fig 4B). In leptotene, the average number of RPA foci was significantly higher at 22 °C (115 ± 25.67) compared to the intermediate temperature (105.55 ± 32.57) (Tukey’s test, p-value < 0.01; Fig 4B), but no significant differences were observed at 30 °C. Late zygotene showed a similar trend, with 68.10 ± 22.92 at 20 °C and 59.47 ± 25.14 at 28 °C (Tukey’s test, p-value < 0.01; Fig 4B).

Interestingly, we observed temperature-dependent variation in both the intra-individual levels of DSB formation and the dynamics of DSB replacement. Notably, nucleus-to-nucleus variability was evident at both low and high temperatures, but not at 28 °C—condition approximating the physiological optimum (S2 Fig). At this temperature (28 °C), no significant differences were found in the number of RPA foci per cell across individuals at any analyzed cell stage (T-test, p > 0.05), although nucleus-to-nucleus variability was still high for one of the individuals (S2 Fig). In contrast, significant differences emerged at both elevated and reduced temperatures in specific cell stages (T-test, p < 0.05).

In addition, we also detected that the dynamics of RPA replacement as prophase I progressed varied among temperatures, with significantly higher average numbers of RPA foci per cell in the pachytene phase at 20 °C (42.92 ± 31.04) and 30 °C (44.10 ± 27.39) compared to 28 °C (19 ± 30.35) (Tukey’s test, p-value < 0.0001; Fig 4B). This pattern was consistent for both individuals analyzed for each temperature (S2 Fig). Altogether, our results show that the number of DSBs in later stages of prophase I (pachytene) are elevated at both high (30 °C) and low temperatures (20 °C and 22 °C) relative to the physiological temperature of 28 °C.

## Discussion

Meiotic recombination is shaped by a range of factors, including evolutionary history across taxa, the structural organization of meiotic chromosomes (e.g., DNA loop size and SC length), and chromosome reorganizations [32,61,62]. While studies in model organisms have shown that extreme temperatures can modulate recombination frequency [5–7], it has remained unclear whether this phenomenon is conserved in ectotherm vertebrates. Here, we demonstrate that temperature influences meiotic recombination in Guibé’s ground gecko (*P. guibeae*), providing one of the few insights into this process in reptiles and underscoring the intricate relationship between environmental conditions and genetic regulation.

### Temperature modulates both CO and DSBs patterns in an ectotherm vertebrate

Our study diverges from previous research by focusing on an ectothermic vertebrate. While earlier studies in *Drosophila* [43,44,63], *Arabidopsis thaliana* [47] and budding yeast [64] have reported an increased meiotic recombination rate at both high and low temperatures, resulting in a U-shaped curve, our work is among the first to explore this pattern in reptiles. Specifically, we detected an increase in the number of COs at both high and low temperatures during the pachytene stage, highlighting the plasticity of meiotic recombination in response to thermal stress and suggesting a conserved adaptive mechanism across diverse taxa.

We also found that the frequency of DSBs followed a similar U-shaped response to temperature at later stages of prophase I (pachytene), with elevated levels in both low and high temperatures relative to the physiological optimum. This suggests either a shared temperature-sensitive mechanism regulating both DSBs and COs along the chromosomal axes, or a cascading effect whereby early events such as DSB induction influence downstream processes like CO designation and formation. During prophase I, chromosomes are organized into large DNA loops attached to the lateral elements of the SC, a proteinaceous structure that facilitates the repair of DSBs and allows recombination in close association with the chromosome axis [54,65–68]. Evidence in mammalian species suggests that DNA loops are organized in a fixed density along chromosomes in a way that loop size and axis length are inversely correlated [31,54,66,69]. Moreover, the number of DSBs has been shown to be proportional to chromosome axis length [70]. Therefore, the interrelationship between DSBs and COs can be influenced by chromosome number and size [31,71].

Each chromosome arm presents at least one CO, fulfilling the requirement for an obligatory chiasma. However, due to positive interference —where one recombination event suppresses the likelihood of another occurring nearby— smaller chromosomes tend to accumulate fewer COs [72]. In *P. guibeae*, which has a relatively low diploid number (2n = 34), the number of meiotic DSBs observed at leptotene under physiological conditions (~100 per cell) aligns with expectations. This contrasts with eutherian mammals, which exhibit higher levels (between 200 and 300 DSBs per cell in leptotene), while marsupials present lower levels (fewer than 150 DSBs per cell in zygotene) [73].

In this context, it has been suggested that stressful ecological factors not only tend to increase recombination rates but can also increase the frequency of DSBs [47]. In the case of *P. guibeae*, temperature could act by slowing down DSB repair, thereby increasing the number of meiotic COs. This could explain the higher number of DSBs we detected in the later stages of prophase I, which is likely to impact the outcome of COs. Specifically, as more DSBs are produced, more COs are generated.

From an evolutionary perspective, our findings point to a potentially adaptive response in *P. guibeae* to thermal stress. The plasticity of the recombination machinery appears to facilitate this response, consistent with previous reports that ectothermic genotypes exhibit heightened recombination sensitivity to environmental stressors [47]. Our results extend this pattern to reptiles. Furthermore, both DSBs and COs exhibit intra-individual variability (cell-to-cell) and intraspecific (individual-to- individual) variability [21,74], a covariation that may provide an adaptive advantage in changing environments [8]. Hyper-CO gametes are advantageous in times of environmental change, when new genetic combinations may be favored, while hypo-CO gametes are advantageous in periods of environmental stasis, when well-evolved ancestral combinations are conserved. This suggests that covariation has the potential to increase the evolutionary adaptability of species through increased CO variability.

Alternatively, the observed changes in recombination may not reflect an adaptive response per se, but rather a byproduct of biophysical stress imposed on the meiotic machinery under suboptimal thermal conditions [75]. The structural sensitivity of components such as the SC implies that even modest thermal perturbations could disrupt CO dynamics [75]. Indeed, we detected significant changes in CO frequency with a temperature increase of just 2 °C above the physiological norm (30 °C versus 28 °C). These findings underscore the thermal sensitivity of meiotic recombination in *P. guibeae* and suggest the existence of threshold effects, where only certain degrees of temperature deviation elicit measurable impacts. Identifying such thresholds will be key to predicting species-specific responses to environmental change.

### Are CO variation and COI by products of temperature variation?

What mechanisms might underlie the observed patterns? Previous studies have reported variation among species as to whether extreme temperatures result in increased and/or decreased recombination rates, indicating that the meiotic recombination machinery is both flexible and functionally significant in environmental adaptation [5–7]. In this way, temperature changes can exert selective pressure on meiotic proteins, thereby modifying pairing, synapsis and recombination during meiosis I. These modifications can, in turn, shift the conditions for optimal fertility. This plasticity in recombination plays a crucial role in enabling species to maintain reproductive success amid environmental stress.

**Fig 5:**
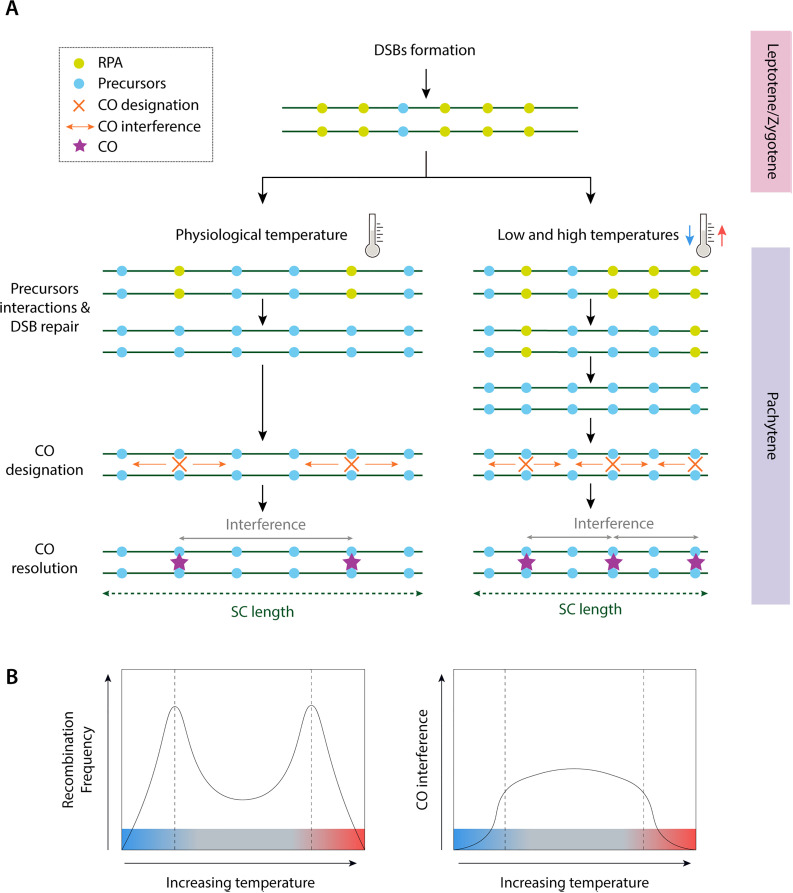
Proposed model for CO formation and resolution in response to temperature variation in reptiles. **(A)** Schematic representation of the process observed in this study. During early stages of prophase I (leptotene/zygotene) double-strand breaks (DSBs) occur. Subsequently, the RPA protein (green dots) binds to single-stranded DNA substrates created after formation of DSBs. DSBs mediate homolog pairing via inter-axis bridge ensembles, which can act as undifferentiated precursors upon by CO-designation [83]. This gives rise to the CO designation with its corresponding interference between COs during CO resolution. Under extreme temperatures (either high or low, *left panel*), two phenomena were observed: (i) decrease in SCs length, and (ii) delayed resolution of RPA foci into CO precursors, hence final COs. This is translated into less interference and thus, high number of final COs. **(B)** Representative graphs showing the distinct responses of COs frequency and interference detected in the present study. Panel on the left represents one of the possible responses to temperature changes a “U-shaped” curve, where CO frequency increases, both above and below the physiological temperature, as previously reported in some model animals, plants and fungi [5]. The panel on the right represents the dynamics of CO interference in response to temperature, which is inversely proportional to CO frequency. Grey boxes indicate temperature ranges that are stable and close to the species’ physiological temperature, while blue and red indicates ranges that are not.

In line with our findings, recent studies in budding yeast [64] have shown that temperature can influence negative supercoils, thereby affecting meiotic COs and chromosome organization. Tan and collaborators [64] found that both increased and decreased temperatures result in shorter meiotic chromosome axes and an increase in COs, likely by modulating CO interference. Consistent with these results, we detected an increase in CO frequency at high and low temperatures that correlated with both a reduction in chromosome axis length and CO interference (Fig 5). This suggests that while the initial formation of DSBs appears largely unaffected by temperature in early stages of meiosis, the final CO designation and resolution are sensitive to thermal variation.

One hypothesis that could explain these patterns is that temperature influences the interaction between CO precursors in late stages of DSB repair, resulting in a high number of CO that are designated and, therefore, resolved. Previous studies [5,64,76] have suggested that the regulation of recombination in response to variation in temperature can be the result of two mechanisms: (i) direct effects on the proteins involved in the formation of COs, or (ii) indirect effects on the structure of chromosome axis. Our results suggest that both processes might be contributing to the observed pattern. Since both chromosome axes and COI are mechanically regulated [53,77,78], the CO increase is more likely a biophysical effect caused by temperature change, i.e., a byproduct of temperature change, as previously suggested [64].

All in all, our results provide new insights into the effects of temperature fluctuations on meiotic recombination in ectothermic species, underscoring the intricate interplay between environmental factors and genetic processes. Further studies will be necessary to elucidate the mechanisms governing the regulation of chromosome axis length and CO resolution in response to temperature.

## Materials and methods

### Ethics statement

Animals were processed in accordance with ethical guidelines approved by Charles University, Prague (UKPRF/28830/2021).

### Experimental setup

A total of 12 adult males were included in the study. Individuals were exposed to different temperatures (20 °C, 22 °C, 24 °C, 26 °C, 28 °C and 30 °C) for seven days prior to the gonad extraction. In accordance with the 3Rs principle (Replacement, Reduction and Refinement), two individuals for each temperature conditions were subjected to study.

### Spermatocyte spreads and immunofluorescence

Spermatocytes spreading were prepared from testicular samples as previously described [33,79]. Testes were disaggregated to obtain a cell suspension, incubated for 30 minutes with 1% Lipsol, and then fixed with 4% paraformaldehyde for 2 hours in a humid chamber. Slides were dried, washed twice with PhotoFlo 1% solution, and either processed for immunofluorescence experiments or stored at -20 °C until use.

Immunostaining was performed to detected meiotic proteins in primary spermatocytes using the following primary antibodies: rabbit antibody against SYCP3 (#ab15093, Abcam, 1:100 dilution), rabbit antibody against RPA32/RPA2 (#ab10359, Abcam, 1:100 dilution), mouse antibody against MLH1 (#ab14206, Abcam, 1:50 dilution), human antibody against centromeric proteins (also known as CREST) (#15-235, Antibodies Incorporated, 1:50 dilution). Fluorochrome-conjugated secondary antibodies from Jackson ImmunoResearch Laboratories were used for detection. All antibodies were diluted in PBS-TritonX100 (0.05%). Primary antibodies were incubated overnight at 4 °C in a humid chamber, followed by secondary antibody incubation at 37 °C for 1 hour. After washing the slides twice to remove excess antibodies, DNA was counterstained with antifade solution containing 8 μg/ml DAPI (4′,6′-diamidino-2-phenylindole).

### Microscopy analysis

Cells were captured using an epifluorescence microscope (Zeiss Axioskop) equipped with emission filters and connected to a camera (ProgRes Jenoptik). The ACO XY program (A. Coloma, Open Microscopy) was used for image acquisition. Meiocytes images were merged and analyzed using Adobe Photoshop. For DSBs studies, cells at various prophase I stages (leptotene, early zygotene, late zygotene, pachytene) were captured and RPA foci were quantified. For COs, only pachytene cells were captured and MLH1 foci scored. Cells were classified into different stages of prophase I, following previous descriptions [33].

### Crossover chromosomal distribution and interference analysis

For the chromosomal distribution of COs, only spermatocytes at pachytene stage with well-defined SCs and MLH1 foci were included in the analysis. For each bivalent, the length of the SC and the distance at which the recombination points were located were analyzed using Micromeasure 3.3 [80] as previously described [29,31,32]. The chromosome position of each MLH1 focus was recorded as a relative position (obtaining a percentage of position with respect to the total SC) and the total length if the SC (expressed in μm) per cell. We calculated the absolute length (in μm), the mean number of MLH1 foci/cell and the CO density (MLH1 μm^-1^) for each autosomal chromosome and temperature. With the acquired data, cumulative CO frequencies were calculated and compared for each temperature by chromosome type (metacentric versus acrocentric). In the case of metacentric chromosomes both the p arm and the q arm were analyzed separately and plotted together.

COI was quantified using two approaches, by analyzing the gamma distribution [56,81] using Rstudio and coefficient of coincidence (CoC) using MADpattern v1.1 [8,57]. For gamma analysis, distributions were fitted using maximum likelihood methods using the fitdistrplus function (of the MASS package from Rstudio) [82]. For CoC analysis, each chromosome arm was scaled to 100% of its total length and divided into 20 segments for CO scoring. For each pair of segments, the observed frequency of double COs (chromosome arms with a CO in both segments) was compared to the expected frequency, determined by multiplying the individual CO frequencies of each segment. The CoC, representing the ratio of observed to expected double COs, was plotted relative to the normalized distance between segments.

### Statistical analysis

Statistical analyses and corresponding p-values were performed in Rstudio, being reported within each plot or detailed in the respective Fig legends. To assess differences in CO frequency, a two-tailed t-test was used to compare the number of MLH1 foci per cell across temperature treatments. A Chi-square test was employed to evaluate the distribution of MLH1 foci per SC. For comparisons involving prophase I stages, temperature conditions, and gamma distribution parameters, Tukey’s HSD test was applied. To analyze cumulative CO frequency along the SC, the Kolmogorov–Smirnov test was used to compare distribution patterns. A significance threshold of p < 0.05 was applied to all tests.

All boxplots were generated using RStudio. In these plots, center lines represent the median, box limits correspond to the interquartile range (25th and 75th percentiles), and whiskers extend to the most extreme data points within 1.5 times the interquartile range from the lower and upper quartiles.

## Supporting information

S1 Table COs in *P. guibeae* for each synaptonemal complex (SC) for individuals (n = 2) treated at 28 °C. Data obtained from a total of 40 spermatocytes.
(XLSX)

S2 Table COs in *P. guibeae* for each synaptonemal complex (SC) for individuals (n = 2) treated at 20 °C. Data obtained from a total of 50 spermatocytes.
(XLSX)

S3 Table COs in *P. guibeae* for each synaptonemal complex (SC) for individuals (n = 2) treated at 30 °C. Data obtained from a total of 50 spermatocytes.
(XLSX)

S4 Table Chromosome relative positions of MLH1 foci per chromosome and temperature, expressed as percentage of chromosomal axes length for each arm. T- test refers to the comparisons with same chromosomes at 28 °C (***p-value < 0.001, **p-value < 0.01). A total of 140 cells were analyzed, distributed as follows: n = 50 cells for 20 °C, n = 40 cells for 28 °C, n = 50 cells for 30 °C.
(XLSX)

S5 Table Gamma values were determined for synaptonemal complexes (SCs) with two MLH1 foci for each temperature. Gamma values between the first and second MLH1 foci in SCs with two MLH1 foci are represented. A total of 140 cells were analyzed, distributed as follows: n = 50 cells for 20 °C, n = 40 cells for 28 °C, n = 50 cells for 30 °C.
(XLSX)

S1 Fig Boxplots represent the number of MLH1 foci per cell for each temperature, separated by individuals (#1 and #2). The boxplots display median values (center line), mean values (dot), and standard deviation (± SD). Violin plots illustrate data pooled from both individuals. A total of 1,169 cells were analyzed across six temperatures: n = 91 cells for 20 °C individual #1, n = 84 cells for 20 °C individual #2, n = 110 cells for 22 °C individual #1, n = 97 cells for 22 °C individual #2, n = 94 cells for 24 °C individual #1, n = 92 cells for 24 °C individual #2, n = 76 cells for 26 °C individual #1, n = 71 cells for 26 °C individual #2, n = 115 cells for 28 °C individual #1, n = 115 cells for 28 °C individual #2, and n = 113 cells for 30 °C individual #1, n = 111 cells for 30 °C individual #2. No statistically significant differences were found between individuals at any treatment temperature (T-test, p-value > 0.05). ns: not significant.
(TIF)

S2 Fig Boxplots representing the number of RPA foci per cell detected at leptotene, early zygotene, late zygotene and pachytene for each temperature, separated by individuals (#1 and #2). Each boxplot displays the median value (center line), mean value (dots), and standard deviation (± SD). Violin plots illustrate the pooled data from both individuals. A total of 1,195 cells were analyzed across five temperatures: n = 162 cells for 20 °C individual #1, n = 104 cells for 20 °C individual #2, n = 103 cells for 22 °C individual #1, n = 105 cells for 22 °C individual #2, n = 96 cells for 26 °C individual #1, n = 137 cells for 26 °C individual #2, n = 100 cells for 28 °C individual #1, n = 118 cells for 28 °C individual #2, and n = 164 cells for 30 °C individual #1, n = 106 cells for 30 °C individual #2. A minimum of 25 cells were analyzed for each cell stage, individual and temperature. Statistical analyses were performed using the T-test, comparing both individuals from each temperature (ns: not significant, *p-value < 0.05, **p-value < 0.01, ***p-value < 0.001).
(TIF)

S1 Data Tables containing the raw data underlying graphs displayed in main and supporting Figs. (XLSX)
