# Supplementary figures and images for: Recombination plasticity in response to temperature variation in reptiles
Source: PLoS Genet. 2025 Aug 4;21(8):e1011772. doi: 10.1371/journal.pgen.1011772 (PMC12342296; doi:10.1371/journal.pgen.1011772)

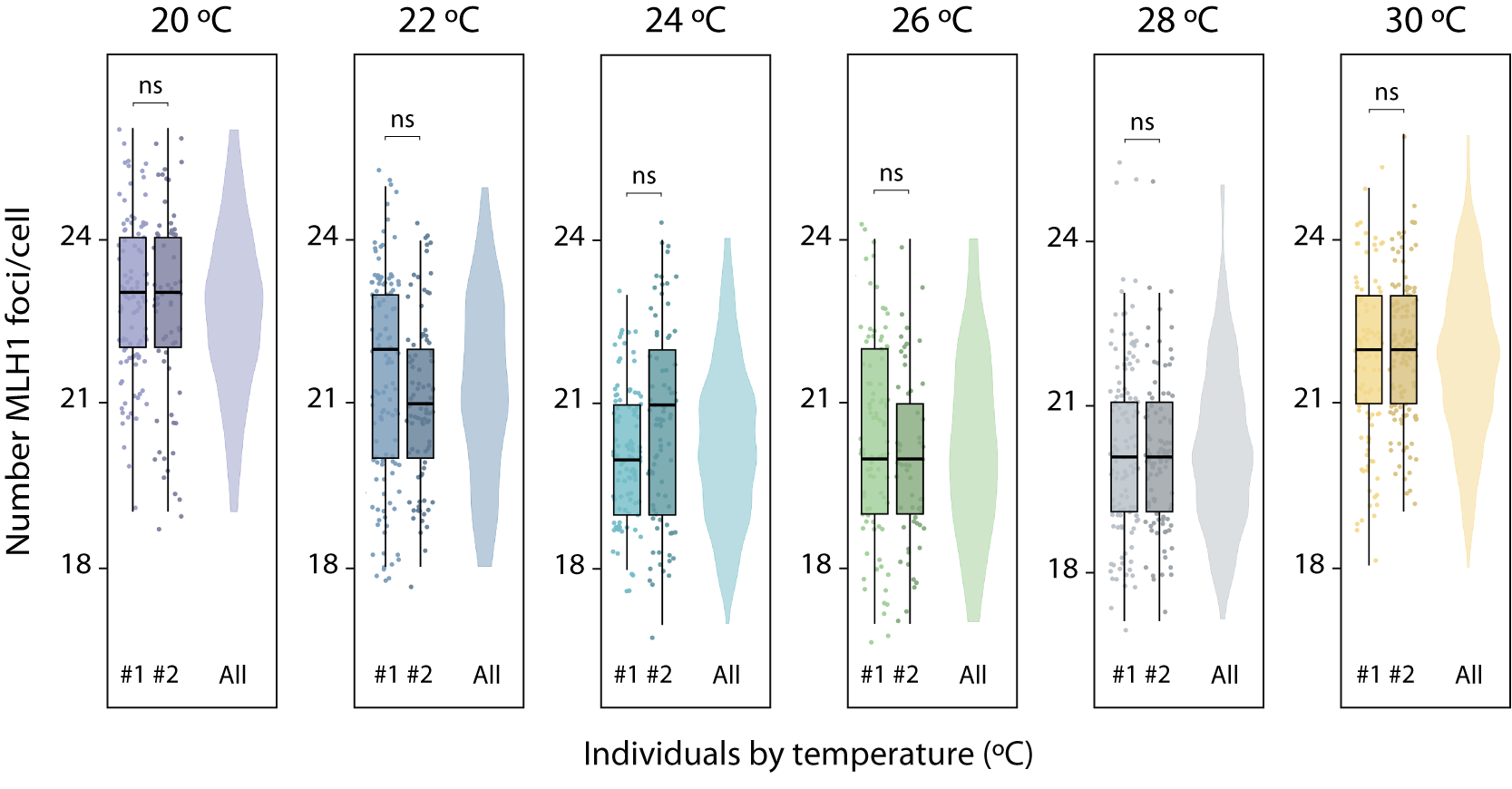

Supplement: S1 Fig — The boxplots display median values (center line), mean values (dot), and standard deviation (± SD). Violin plots illustrate data pooled from both individuals. A total of 1,169 cells were analyzed across six temperatures: n = 91 cells for 20 °C individual #1, n = 84 cells for 20 °C individual #2, n = 110 cells for 22 °C individual #1, n = 97 cells for 22 °C individual #2, n = 94 cells for 24 °C individual #1, n = 92 cells for 24 °C individual #2, n = 76 cells for 26 °C individual #1, n = 71 cells for 26 °C individual #2, n = 115 cells for 28 °C individual #1, n = 115 cells for 28 °C individual #2, and n = 113 cells for 30 °C individual #1, n = 111 cells for 30 °C individual #2. No statistically significant differences were found between individuals at any treatment temperature (T-test, p-value > 0.05). ns: not significant. (TIF) [file pgen.1011772.s006.tif]

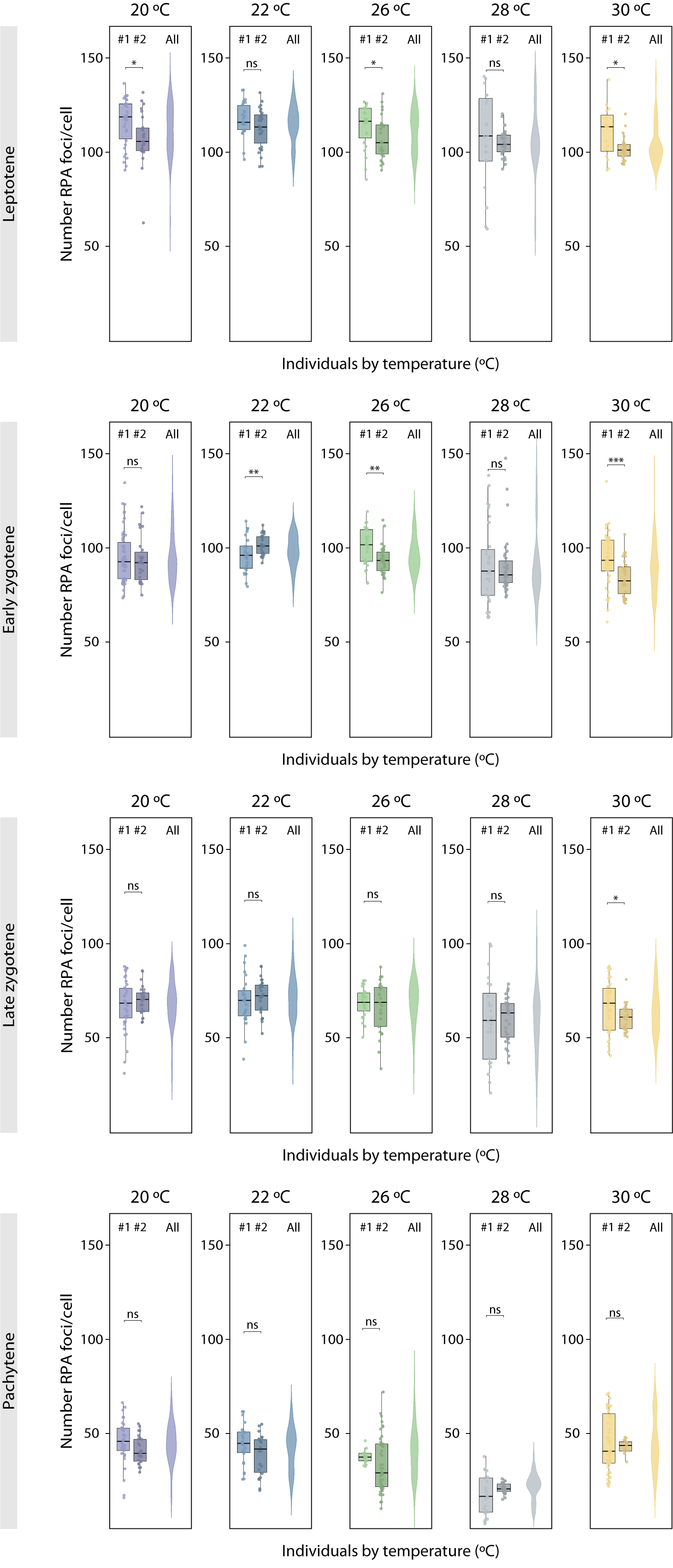

Supplement: S2 Fig — Each boxplot displays the median value (center line), mean value (dots), and standard deviation (± SD). Violin plots illustrate the pooled data from both individuals. A total of 1,195 cells were analyzed across five temperatures: n = 162 cells for 20 °C individual #1, n = 104 cells for 20 °C individual #2, n = 103 cells for 22 °C individual #1, n = 105 cells for 22 °C individual #2, n = 96 cells for 26 °C individual #1, n = 137 cells for 26 °C individual #2, n = 100 cells for 28 °C individual #1, n = 118 cells for 28 °C individual #2, and n = 164 cells for 30 °C individual #1, n = 106 cells for 30 °C individual #2. A minimum of 25 cells were analyzed for each cell stage, individual and temperature. Statistical analyses were performed using the T-test, comparing both individuals from each temperature (ns: not significant, *p-value < 0.05, **p-value < 0.01, ***p-value < 0.001). (TIF) [file pgen.1011772.s007.tif]
